# Clinical Outcome Prediction of Early Brain Injury in Aneurysmal Subarachnoid Hemorrhage: the SHELTER-Score

**DOI:** 10.1007/s12028-023-01879-y

**Published:** 2023-11-29

**Authors:** Björn B. Hofmann, Daniel M. Donaldson, Milad Neyazi, Yousef Abusabha, Kerim Beseoglu, Daniel Hänggi, Jan F. Cornelius, Igor Fischer, Sajjad Muhammad

**Affiliations:** 1 Department of Neurosurgery, Medical Faculty and University Hospital Düsseldorf, Heinrich-Heine-University Düsseldorf, Moorenstraße 5, Düsseldorf, Germany; 2 Department of Neurosurgery, International Neuroscience Institute, Hannover, Germany; 3 Department of Neurosurgery, King Edward Medical University, Lahore, Pakistan; 4 Department of Neurosurgery, University of Helsinki and Helsinki University Hospital, Helsinki, Finland

**Keywords:** Aneurysmal subarachnoid hemorrhage, Brain injury, Vascular, Early brain injury, Intensive care, Outcome study

## Abstract

**Background:**

Despite intensive research on preventing and treating vasospasm and delayed cerebral ischemia in aneurysmal subarachnoid hemorrhage (aSAH), mortality and morbidity rates remain high. Early brain injury (EBI) has emerged as possibly the major significant factor in aSAH pathophysiology, emphasizing the need to investigate EBI-associated clinical events for improved patient management and decision-making. This study aimed to identify early clinical and radiological events within 72 h after aSAH to develop a conclusive predictive EBI score for clinical practice.

**Methods:**

This retrospective analysis included 561 consecutive patients with aSAH admitted to our neurovascular center between 01/2014 and 09/2022. Fourteen potential predictors occurring within the initial 72 h after hemorrhage were analyzed. The modified Rankin Scale (mRS) score at 6 months, discretized to three levels (0–2, favorable; 3–5, poor; 6, dead), was used as the outcome variable. Univariate ordinal regression ranked predictors by significance, and forward selection with McFadden’s pseudo-*R*^2^ determined the optimal set of predictors for multivariate proportional odds logistic regression. Collinear parameters were excluded, and fivefold cross-validation was used to avoid overfitting.

**Results:**

The analysis resulted in the Subarachnoid Hemorrhage Associated Early Brain Injury Outcome Prediction score (SHELTER-score), comprising seven clinical and radiological events: age (0–4 points), World Federation of Neurosurgical Societies (0–2.5 points), cardiopulmonary resuscitation (CPR) (2 points), mydriasis (1–2 points), midline shift (0.5–1 points), early deterioration (1 point), and early ischemic lesion (2 points). McFadden’s pseudo-*R*^2^ = 0.339, area under the curve for death or disability 0.899 and 0.877 for death. A SHELTER-score below 5 indicated a favorable outcome (mRS 0–2), 5–6.5 predicted a poor outcome (mRS 3–5), and ≥ 7 correlated with death (mRS 6) at 6 months.

**Conclusions:**

The novel SHELTER-score, incorporating seven clinical and radiological features of EBI, demonstrated strong predictive performance in determining clinical outcomes. This scoring system serves as a valuable tool for neurointensivists to identify patients with poor outcomes and guide treatment decisions, reflecting the great impact of EBI on the overall outcome of patients with aSAH.

**Supplementary Information:**

The online version contains supplementary material available at 10.1007/s12028-023-01879-y.

## Introduction

Despite intensive efforts to improve the diagnosis and treatment of aneurysmal subarachnoid hemorrhage (aSAH), it remains a highly fatal and morbid disease [1]. Besides treatment of the bleeding aneurysm, intensive care therapy for accompanying complications is crucial for the patient’s outcome [1–4]. Although recent advancements in intensive care management have made significant strides, they have not yet resulted in a breakthrough for reducing the morbidity associated with aSAH-specific complications. One reason for this is a still insufficient understanding of the exact pathophysiological processes during and after acute subarachnoid hemorrhage. Over time, two pathophysiological phases have been defined after aSAH in which different processes can occur to varying degrees [5]. The first phase, defined as the first 72 h after hemorrhage, is the phase of early brain injury (EBI) [6–9]. This period is followed by the delayed cerebral ischemia (DCI) phase [10–12]. For a long time, the occurrence of vasospasm in the DCI phase was believed to be the main determinant of patient outcome [13–15]. Thus, research in recent decades has primarily focused on diagnosis and prevention of cerebral vasospasm leading to DCI. However, despite these efforts, mortality and morbidity of aSAH have not significantly improved over the years. In fact, it has been shown that, contrary to popular belief, the occurrence of vasospasm might be a frequent and important factor in DCI, but DCI can develop even in the absence of angiographic vasospasm [5, 16–18]. For instance, a systematic review of several studies concluded that even when the rate of vasospasm was pharmacologically controlled, no improved clinical outcome in patients could be observed [5, 19]. Furthermore, a recent clinical trial showed that the endothelin-1 receptor antagonist clazosentan reduced angiographically detectable vasospasm but did not significantly improve functional outcome [16–18].

Growing knowledge and understanding from experimental aSAH research demonstrate that pathophysiological processes in the early phase after bleeding can set the stage for subsequent long-term complications and thus have a major impact on the patient’s outcome [5, 9, 20]. In short, the inflammatory cascade initiated during EBI leads to microvascular dysfunction, blood–brain barrier disruption, and formation of microthrombosis, which affects the clinical outcome by sustaining an inflammatory response [5, 9, 21, 22].

In this study, we aimed to identify clinical and radiological events occurring in the first 72 h after hemorrhage that predict outcome and mortality. The objective was to develop and internally validate an EBI score to predict the clinical outcome of the patients after aSAH.

## Methods

### Ethical Statement

All procedures performed in studies involving human participants adhered to the ethical standards of the institutional committee and the 1964 Helsinki declaration and its later amendments. The study was approved by the local ethics committee of the Medical Faculty of the Heinrich-Heine University, Düsseldorf, Germany (study ID: 2022–2292), and the need for written informed consent was waived due to the retrospective study design. The article was prepared in accordance with the Strengthening the Reporting of Observational Studies in Epidemiology guidelines.

### Inclusion Criteria

The study included all patients with subarachnoid hemorrhage admitted to the neurovascular center from 01/2014 to 09/2022 who met the following inclusion criteria: (1) subarachnoid hemorrhage confirmed by initial nonenhanced computed tomography (CT) and (2) verification of an aneurysm in the course by means of CT angiography and/or digital subtraction angiography. Patients were excluded from the study if the modified Rankin Scale (mRS) after 6 months was not available or could not be collected retrospectively, or if 6 months had not yet passed after bleeding at the time of evaluation.

### Subarachnoid Hemorrhage Management

All patients who reached the clinic, regardless of their hemorrhage severity or World Federation of Neurosurgeons (WFNS) grading, were included in the study. All patients received curative therapy except if initial CT angiography and CT perfusion imaging revealed a perfusion arrest of the brain, or in some elderly patients with fulminant hemorrhage (high Fischer and WFNS grade) with a clearly communicated patient will, no curative but palliative therapy was administered (in total, 27 patients did not receive treatment of the aneurysm). The treatment of patients with aSAH follows an in-house treatment guideline, which has been described in detail before [23]. In short, patients were admitted to the neurosurgical intensive care unit after aSAH was detected. With a Glascow Coma Scale (GCS) of 13 and higher, patients were closely monitored with as little manipulation as possible (no-touch technique) until aneurysm occlusion. If the GCS was 12 or lower, anesthesia, intubation, and surgical placement of an external ventricular drain for intracranial pressure monitoring were performed. If possible, the aneurysm was subsequently treated within 24 h after admission during regular working hours after interdisciplinary consultation either surgically or endovascularly. If immediate relief and hemorrhage evacuation were necessary due to larger intraparenchymal blood components, the aneurysm was clipped in the same emergency operation. Patients received a follow-up cranial CT scan with CT angiography and CT perfusion imaging 6 h after surgery/intervention. Subsequently, control imaging by cranial CT and CT perfusion was performed approximately every 3 days (i.e., on day 4, 7, 11, and possibly 14 after admission). Radiographic or clinical evidence of a perfusion delay was managed with induced hypertension and, in selected cases, by intraatrial administration of nimodipine.

### Survey of the Parameters

Patient characteristics, radiological and clinical data of the patients with aSAH were retrospectively collected from the electronic medical record. The following parameters were determined for the development of the EBI score based on available medical documentation and radiographic imaging: (1) CPR (prehospital) of the patient, mydriasis, unilateral with consequent anisocoria or bilateral dilatation of the pupils with decreased light response at any time throughout the first 72 h after bleeding (requirement 1) clear pronouncement documented by a neurosurgeon; (2) in cases of suspected drug-induced mydriasis, it was only considered positive if there were pathologically elevated intracranial pressure values or if cranial imaging showed signs of herniation; (3) volume in milliliters of intraparenchymal hemorrhage on initial CT imaging; (4) presence of midline shift on initial CT imaging; (5) early ischemia (excluding ischemia if clearly associated with clipping or coiling) with demarcation within 3 days after hemorrhage on CT imaging; (6) early deterioration, defined as a GCS drop of 2 points without any other cause within 72 h after hemorrhage; (7) aneurysm location (subdivided into the 13 localizations listed in Table 1); and (8) treatment (subdivided into the seven treatment options listed in Table 1).

**Table 1:** Patients characteristics

Characteristic	Patients (*N* = 561)
Sex	
Female	382
Male	179
Age	
Mean ± SD	56.26 ± 13.47
Minimum	20
Maximum	93
GCS	
3	109
4	21
5	13
6	14
7	14
8	15
9	13
10	13
11	15
12	18
13	22
14	94
15	200
WFNS grade	
1	200
2	92
3	24
4	87
5	158
Fisher score	
1	19
2	30
3	438
4	74
Modified Fisher score	
0	14
1	40
2	61
3	122
4	324
Aneurysm location	
MCA	138
ACOM	210
ICA	39
PcaA	25
PCOM	55
BA	44
VA	13
PICA	19
SCA	1
PCA	8
AICA	1
A. choroidea anterior	5
Other	3
Treatment	
Clipping	310
Trapping	7
Wrapping	2
Coiling	190
Flowdiverter	24
Coiling and clipping	1
None	27
mRS 6 months	
0	130
1	109
2	37
3	46
4	35
5	89
6	115

*ACOM*, anterior communicating artery, *AICA*, anterior inferior cerebellar artery, *BA*, basilar artery, *GCS*, Glasgow Coma Scale, *ICA*, internal carotid artery, *MCA*, middle cerebral artery, *mRS*, modified Rankin Scale, *PcaA*, pericallosal artery, *PCOM*, posterior communicating artery, *PICA*, posterior inferior cerebellar artery, *SCA*, superior cerebellar artery, *SD*, standard deviation, *VA*, vertebral artery, *WFNS*, World Federation of Neurosurgical Societies

### Definition of aSAH Grading and Outcome Measures

The severity of aSAH was assessed by initial WFNS score, which was determined before any anesthesia and intubation in case of reduction of GCS. The outcome was assessed by the mRS at discharge and 6 months after bleeding, assessed in a clinical follow-up.

### Study Design and Statistical Analysis

The data set was first randomly divided into a training and a test set in a 2:1 ratio. Fourteen potential predictors including age, sex, aneurysm location, therapy, GCS, WFNS score, Fisher score, modified Fisher score, mydriasis, early ischemia, early deterioration, intraparenchymal blood, midline shift, and CPR were selected based on the currently known predictors and current knowledge of pathophysiological processes. The mRS score at 6 months, categorized into three levels (0–2, favorable; 3–5, disability; 6, dead), was chosen as the outcome variable.

The first step in the analysis involved using univariate ordinal regression (proportional odds logistic regression [POLR]) to rank the predictors by significance. Next, forward selection was performed, starting with the most significant predictor and using McFadden’s pseudo-R^2^ as the optimality criterion, to determine the optimal set of predictors for multivariate POLR. Fivefold cross-validation was used to avoid overfitting. The final model was tested and applied to the test set.

The parameters found by POLR were real numbers with many decimal places, which were impractical for direct use by humans. Therefore, the proposed score was derived by rounding and manually adjusting the parameters on the basis of statistical and clinical considerations. The authors believe that the proposed score’s ease of use outweighs its slightly reduced predictive performance. The calculations were performed by using Python 3.9.7 and the NumPy, SciPy, and Statsmodels packages.

## Results

### Patient Characteristics

After applying the exclusion criteria to the initially screened 989 patients with subarachnoid hemorrhage, 402 patients were excluded because they had no evidence of an aneurysm, and 26 patients were excluded because they lacked 6-month follow-up data. This left a total of 561 patients who were included in the current study cohort (details are depicted in Supplementary Fig. 1). The mean age of the patients was 56 ± 13 years, and 382 (68%) of patients were women. Table 1 provides detailed information on the patient characteristics. The complete set was randomly split into a training set, containing 374 patients, and a test set, with 187 patients.

### Clinical and Radiological Features of EBI Phase

**Fig. 1:**
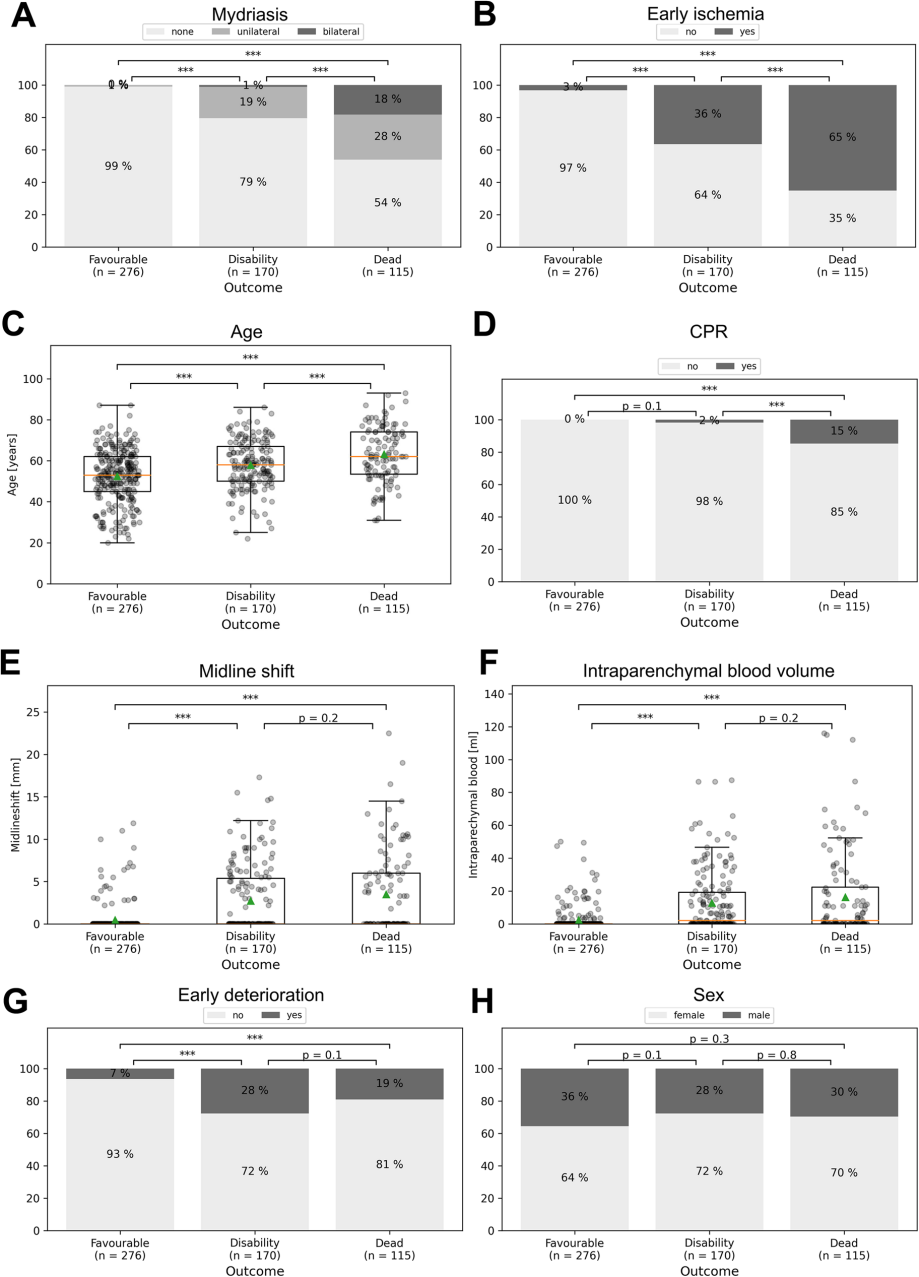
EBI-phase parameters. Representation of the EBI-phase parameters in bar graphs (dichotomized or trichotomized numbers) or boxplots (real numbers) for mydriasis (**a**), early ischemia (**b**), age (**c**), cardiopulmonal resuscitation (CPR) (**d**), midline shift (**e**), intraparenchymal blood volume (**f**), early deterioration (**g**), and sex (**h**). Significance was defined as *p* value < 0.05 (**p < 0.01, ***p < 0.001) for comparison between the different outcome groups; mRS score at 6 months categorized into three levels (0–2, favorable; 3–5, disability; 6, dead). EBI, early brain injury

In the EBI phase, we observed noteworthy differences among the three trichotomized outcome groups. Specifically, patients with mydriasis or early deterioration and those with higher age had significantly poorer outcomes and higher mortality rates (Fig. 1a, b, and c). Moreover, there was a trend toward increased rates of resuscitation in the group of patients who died, although this trend did not reach statistical significance (Fig. 1d). For the parameters of midline shift, intraparenchymal blood volume, and early deterioration, we found a significant increase in patients in the disability group, but no significant differences between the disability and deceased groups (Fig. 1e, f, and g). Notably, we observed an even distribution of sex across all outcome groups, with no significant differences (Fig. 1h). To further assess the predictive value of each EBI parameter, we constructed receiver operating characteristic curves (Supplementary Fig. 2).

### EBI Score Development (The so-Called Subarachnoid Hemorrhage Associated Early Brain Injury Outcome Prediction Score)

To develop the EBI score, we first performed univariate ordinal regression of the EBI parameters, as described in the Material and Methods section. The results are shown in Supplementary Table 1.

The forward selection analysis excluded the variable intraparenchymal blood volume due to its high collinearity with midline shift (as shown in Supplemental Fig. 4). Additionally, the analysis also revealed high collinearity between the WFNS grade and the GCS score, resulting in the exclusion of the GCS score from the forward selection because the WFNS score could be used without further modification to construct the EBI score. Fivefold cross-validation was used to avoid overfitting (McFadden’s pseudo R^2^ = 0.316). The final model was tested on the test set of 187 patients and showed slightly worse performance (R^2^ = 0.332) than on the training set (R^2^ = 0.357), as could be expected. The performance on the original, unified set was R^2^ = 0.349. We optimized the numerical values of the score, as described in the Material and Methods section, resulting in the Subarachnoid Hemorrhage Associated Early Brain Injury Outcome Prediction score (SHELTER-score), which is shown in Table 2.

**Table 2:** Multivariant SHELTER-score to predict patient outcome

EBI-phase parameter		SHELTER-score *p*oints
Age	< 20	0
	20–39	1
	40–59	2
	60–79	3
	≥ 80	4
WFNS grade	0	0
	1	0.5
	2	1
	3	1.5
	4	2
	5	2.5
Cardiopulmonary resuscitation	Ensued	2
Mydriasis	Anisocoria	1
	Bilateral	2
Midline shift	> 10 mm	0.5
	> 20 mm	1
Early deterioration	Present	1
Early ischemia	Present	2

		**= sum score**
**SHELTER-score ≤ 4.5**	**Good outcome (favorable)**	
**5 ≤ SHELTER-score ≤ 6.5**	**Poor outcome (disability)**	
**SHELTER-score ≥ 7**	**Dead**	

Good outcome with 6-month mRS = 0–2; poor outcome with 6-month mRS = 3–5; dead with 6-month mRS = 6

*EBI*, early brain injury, *mRS*, modified Rankin Scale, *SHELTER-score*, Subarachnoid Hemorrhage Associated Early Brain Injury Outcome Prediction score, *WFNS*, World Federation of Neurosurgical Societies

### SHELTER-Score Performance and Internal Validation

**Fig. 2:**
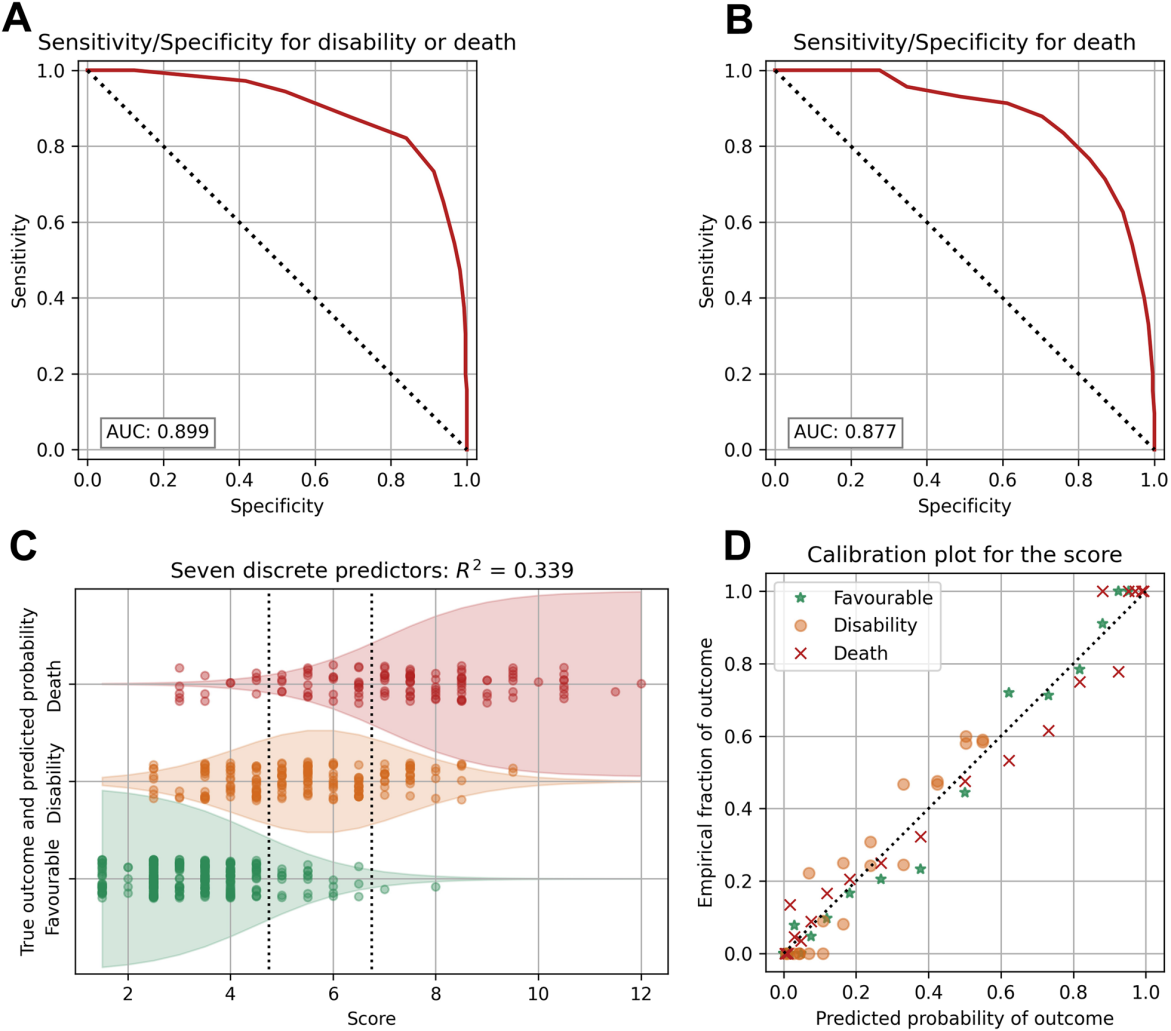
SHELTER-score performance. Predictive value of the SHELTER-score for disability or death (**a**) and death after 6 months (**b**) using receiver operating characteristics (ROC) curves. (**c**) Visual representation of the probability of individual outcomes and the correspondence between the empirical proportions of patients in three groups and the predicted probability (**d**) is depicted. mRS score at 6 months categorized into three levels (0–2, favorable; 3–5, disability; 6, dead). AUC, area under the curve, mRS, modified Rankin Scale, SHELTER-score, Subarachnoid Hemorrhage Associated Early Brain Injury Outcome Prediction score

The performance of the final SHELTER-score, measured on the unified set, was R^2^ = 0.339, *c*-index = 0.872, *p* < 0.001. The area under the curve was 0.899 for predicting disability or death after 6 months and 0.877 for predicting death (Fig. 2a, b). We also examined the distribution of the SHELTER-score in relation to the trichotomized outcome and presented the results in Table 3. Additionally, the probability of individual outcomes was visually represented in Fig. 2c, and the correspondence between the empirical proportions of patients in the three outcome groups and the predicted probability is shown in Fig. 2d.

**Table 3:** Related outcome to each risk point of the SHELTER-score

EBI-score	Good outcome (favorable) (%)	Poor outcome (disability) (%)	Death (%)
1.5	95.26	4.34	0.41
2	92.4	6.92	0.67
2.5	88.08	10.82	1.1
3	81.76	16.44	1.8
3.5	73.11	23.96	2.93
4	62.25	33.01	4.74
4.5	50	42.41	7.59
5	37.75	50.33	11.92
5.5	26.89	54.86	18.24
6	18.24	54.86	26.89
6.5	11.92	50.33	37.75
7	7.59	42.41	50
7.5	4.74	33.01	62.25
8	2.93	23.96	73.11
8.5	1.8	16.44	81.76
9	1.1	10.82	88.08
9.5	0.67	6.92	92.41
10	0.41	4.34	95.26
10.5	0.25	2.68	97.07
11	0.15	1.65	98.2
11.5	0.09	1.01	98.9
12	0.06	0.61	99.33

mRS score at 6 months categorized into three levels (0–2, favorable; 3–5, disability; 6, dead)

*EBI*, early brain injury, *mRS*, modified Rankin Scale, *SHELTER-score*, Subarachnoid Hemorrhage Associated Early Brain Injury Outcome Prediction score

## Discussion

In this work, we were able to create an EBI score comprising seven key variables, which allowed prediction of the neurological outcome of the patients after aSAH with very high accuracy. We named this EBI prediction score the Subarachnoid Hemorrhage Associated Early Brain Injury Outcome Prediction score (SHELTER-score), which consists of the following independent predictors: age, WFNS, anisocoria, early deterioration, CPR, midline shift, and early ischemia. The SHELTER-score has numerous implications for clinical decision-making and patient care, as it can help clinicians predict the course of the disease and determine appropriate treatment plans. Additionally, the score highlights the importance of EBI events in the pathophysiology of aSAH, emphasizing the need for further research in this area.

In recent years, there has been an increasing understanding of the pathophysiological processes after aSAH. Despite decades of research on cerebral vasospasm, the lack of development of successful treatment options has shifted the focus of research toward detecting early clinical events after subarachnoid hemorrhage to develop novel diagnostic, outcome, and therapeutic tools. The early pathophysiological processes in the phase of EBI have progressively come into focus, whereby a cascade of sterile inflammation following tissue injury after aSAH leads to cellular stress, apoptosis, blood–brain barrier disruption, and microvascular dysfunction, which in turn maintains and even drives an inflammatory response [5, 9, 22]. The SHELTER-score developed in this study therefore focuses on risk factors from the EBI phase and aims to predict the influence of EBI on the neurological outcome of patients with aSAH, independent of the phase of DCI. Through the very high predictive value and accuracy of our SHELTER-score, we see the immense importance of the tissue damage within the EBI for the overall outcome in patients with aSAH.

The parameters used in the SHELTER-score were selected based on the results of forward selection. Our study population showed that age strongly influences patient outcomes, which is consistent with previous studies [24–27]. Elderly patients are more likely to have preexisting conditions that can worsen the severity and duration of complications, leading to a significantly worse outcome [24–27]. To reflect this, our score divides age into five categories with ascending score values. Patients over the age of 80 are assigned the maximum score (4) in the entire score, emphasizing the critical role of age in determining outcomes.

Another well-established predictor of patient outcome after aSAH is the initial neurological condition of the patient, commonly assessed by using the WFNS grading system [28–30]. In our study population, the WFNS grade was found to be the parameter with the highest predictive power, and it was assigned a point value of 0.5 for each consecutive grade in the SHELTER-score.

We also included the EBI parameter of prehospital CPR in our score, as previous studies have shown that aneurysmal hemorrhage can be associated with cardiac arrest requiring CPR in the prehospital setting [31–33]. Our data demonstrate that patients who required CPR generally had worse outcomes than those who did not (Fig. 1d). This may be due, in part, to an initial massive brain damage and maximum intracranial pressure increase that can trigger cardiac arrest [31, 32, 34]. Furthermore, cardiac arrest in the context of aSAH can also mask the underlying bleeding problem and lead to a worse outcome by delaying adequate therapy. In our study population, the prognostic value of CPR was also evident, although only 2 points were attributed to this parameter after weighting in our SHELTER-score.

Another significant neurological symptom in clinical settings is the presence of dilated unilateral or bilateral pupils, which often indicates the need for immediate emergency surgery [35, 36]. Therefore, it is not surprising that unilateral or bilateral mydriasis is also an important predictor of patients’ outcomes in our patient cohort. In clinical practice, bilateral dilated pupils typically indicate the most severe brain or brainstem damage [37, 38]. Our SHELTER-score reflects this condition with an increased score of one point for anisocoria with mydriasis and two points for bilateral mydriasis. It is essential to note that the score takes into account not only the possible initial anisocoria at the time of ictus but also any anisocoria that may occur during the EBI phase.

Anisocoria is typically caused by a unilateral space-occupying process, primarily intraparenchymal hemorrhage or, more rarely, an acute subdural hematoma in the acute phase of aSAH [39, 40]. In the later course (secondarily), brain swelling due to hypoxia leading to severe edema is another reason for a space-occupying process [39]. These ultimately result in a displacement of the remaining intact brain tissue, visible in CT imaging as a midline shift. Therefore, there is a strong collinearity between these space-occupying effects and the occurrence of a midline shift. This collinearity between intraparenchymal blood volume in milliliters and midline shift in millimeters is also evident in our patient cohort (Supplementary Fig. 4), so we included only the stronger prognostic factor, the midline shift, in the SHELTER-score. The midline shift is not only the better prognostic factor but also easier and faster to measure than the amount of bleeding on CT. The increasing predictive value for poor patient outcome with increasing midline shift is reflected in the distinction between a midline shift greater than 10 mm (score: 0.5) and a midline shift greater than 20 mm (score: 1) in our SHELTER-score.

Another parameter that is important to consider when assessing the severity of EBI is early deterioration, which refers to a patient’s clinical decline within the first 72 h without any other identifiable cause [5, 9]. This deterioration is caused by multiple pathophysiological processes that lead to early brain damage, which in turn promotes further damage in the course of the disease [5, 9]. In line with the literature, our study found that early deterioration has a high prognostic value for patient outcome, which is why it was included in our SHELTER-score with a score value of 1.

Early deterioration reflects early brain damage, whereas early ischemia, as the most severe form, has already resulted in demarcated infarcted areas in the brain visible on CT imaging, as a manifestation of tissue hypoxia. The occurrence of early ischemia is heavily weighted in our SHELTER-score, with a score value of 2 given its high predictive accuracy in our study population.

In conclusion, by using the EBI variables, we developed a score with a very high predictive accuracy for patient outcome in the studied population of patients with aSAH. The strong correlation of the SHELTER-score with patient outcome highlights the critical nature of early-phase damage following subarachnoid hemorrhage for the overall disease course. Our SHELTER-score may prove valuable to clinicians in predicting the course of aSAH and making treatment decisions for affected patients with this life-threatening condition.

When interpreting these findings, it’s important to acknowledge that this is a single-center, retrospective study, and the resulting score needs to be replicated and validated in other populations to estimate the generalizability of the SHELTER-score. Furthermore, only the 6-month mRS was used as an outcome variable, and other important outcome parameters such as cognitive status or quality of life of the affected patients were not taken into account in the compilation of the SHELTER-score. Additionally, future studies with a longer follow-up duration would be valuable for providing an even more comprehensive prediction of long-term outcomes. Additionally, preexisting health conditions of the patients were not taken into account and our study examined a specific set of clinical and radiological variables, and there may be additional prognostic factors that were not considered during the development of the scoring system (e.g., lab values). Incorporating these factors could potentially enhance the performance of the score. However, it is crucial to strike a balance between practicality and comprehensiveness. In our study, we prioritized variables that could be readily recorded in a timely manner. This decision was made to ensure the score’s practicality and ease of use in the clinical setting.

## Conclusions

The SHELTER-score, which includes seven clinical and radiological features of EBI (age, WFNS grade, cardiopulmonary resuscitation, mydriasis, midline shift, early deterioration, and early ischemia), has high sensitivity and specificity for predicting clinical outcome. The SHELTER-score is an excellent tool for guiding neurointensivists to identify patients with poor outcomes and aid in treatment decision-making. Our clinical data highlight the significant impact of EBI on the overall outcome of patients with aSAH.

### Supplementary Information

Below is the link to the electronic supplementary material.

Supplementary file 1 (DOCX 700 KB)
