# Supplementary material for: Clinical Outcome Prediction of Early Brain Injury in Aneurysmal Subarachnoid Hemorrhage: the SHELTER-Score
Source: Neurocrit Care. 2023 Nov 29;40(2):438–47. doi: 10.1007/s12028-023-01879-y (PMC10959788; doi:10.1007/s12028-023-01879-y)
Supplement: Supplementary file 1 — (DOCX 700 KB) [file 12028_2023_1879_MOESM1_ESM.docx]

**Clinical Outcome Prediction of Early Brain Injury in Aneurysmal Subarachnoid hemorrhage: The SHELTER Score**

Björn B. Hofmann MD^1^, Daniel M. Donaldson MD^1^, Milad Neyazi MD^1^, Yousef Abusabha MD^1^, Kerim Beseoglu MD, PhD^1^, Daniel Hänggi MD, PhD^1,2^, Jan F. Cornelius MD, PhD^1^, Igor Fischer PhD^1^, Sajjad Muhammad MD, PhD^1,3,4^

^1^ Department of Neurosurgery, Medical Faculty and University Hospital Düsseldorf, Heinrich-Heine-University Düsseldorf, Düsseldorf, Germany

^2^ Department of Neurosurgery, International Neuroscience Institute, Hannover, Germany

^3^ Department of Neurosurgery, King Edward Medical University, Lahore, Pakistan

^4^ Department of Neurosurgery, University of Helsinki and Helsinki University Hospital, Helsinki, Finnland

**Supplemental Files**

Table of contents

Supplemental Figur 1 - Page 2

Supplemental Figur 2 - Page 3

Supplemental Figur 3 - Page 4

Supplemental Figur 4 - Page 5

Supplemental Table 1 - Page 6

**
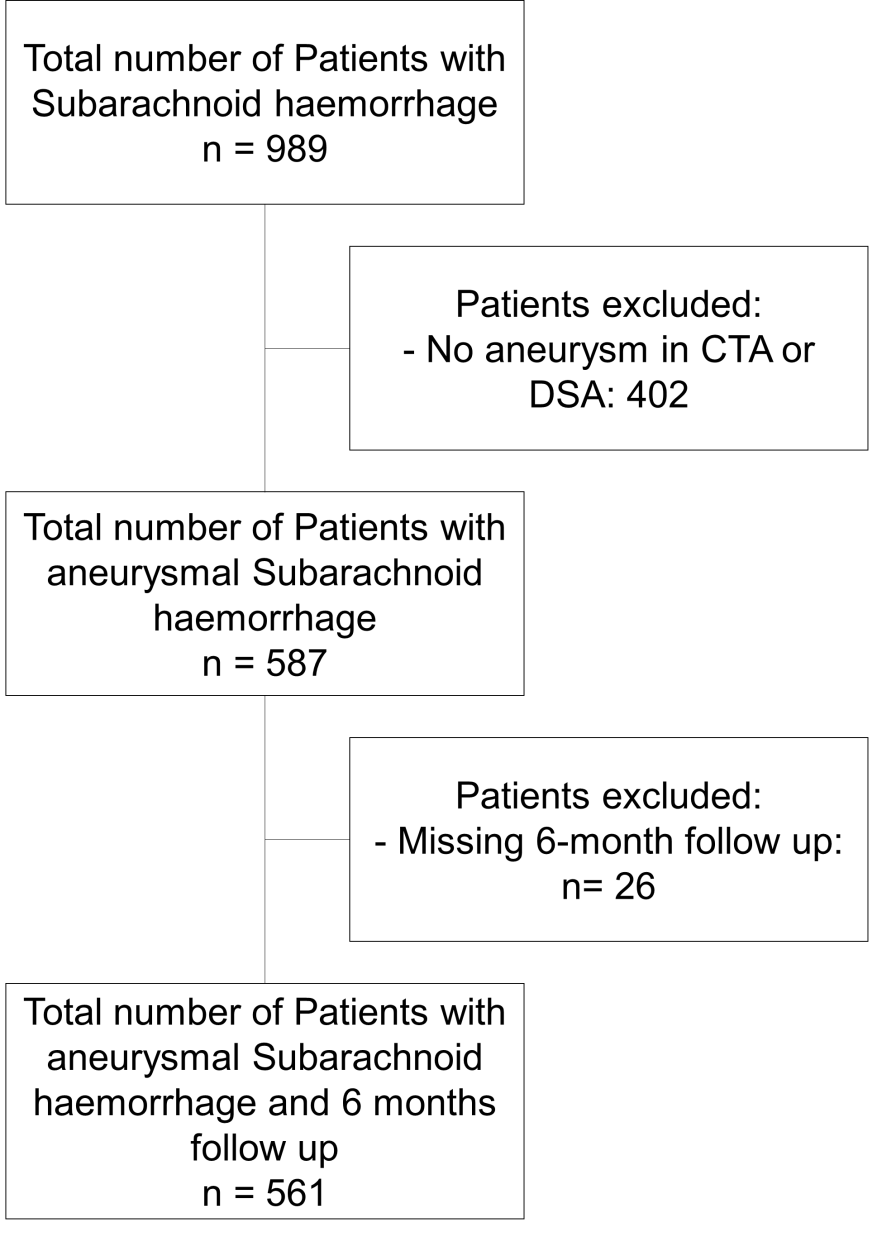
**

**Supplemental Figure 1: Flow diagram of numbers of individuals at each stage of screening**

**
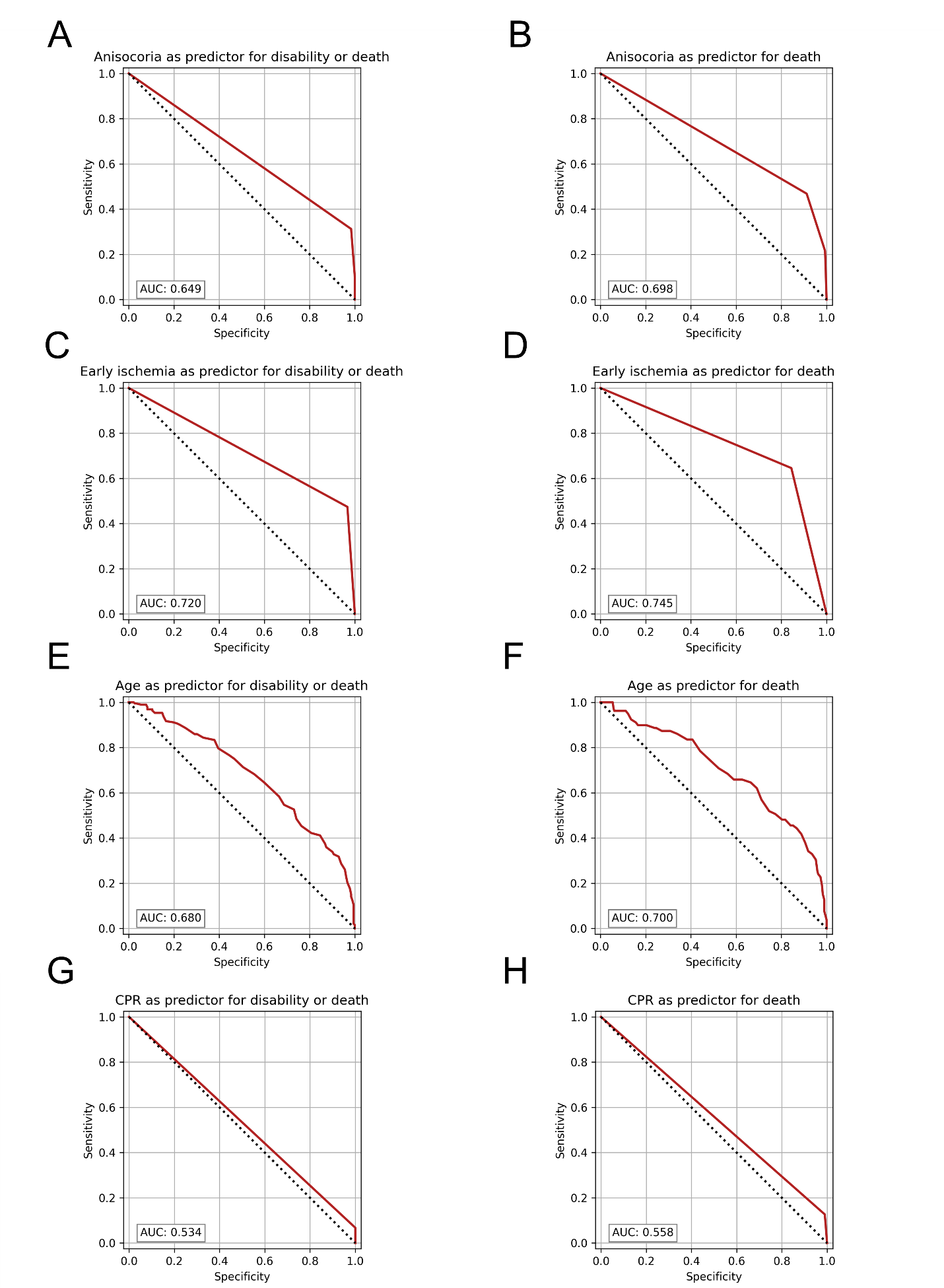
**

**Supplemental Figure 2:** Predictive value of each EBI-parameter for the outcome (6 months mRS) using receiver operating characteristics (ROC) curves.

**
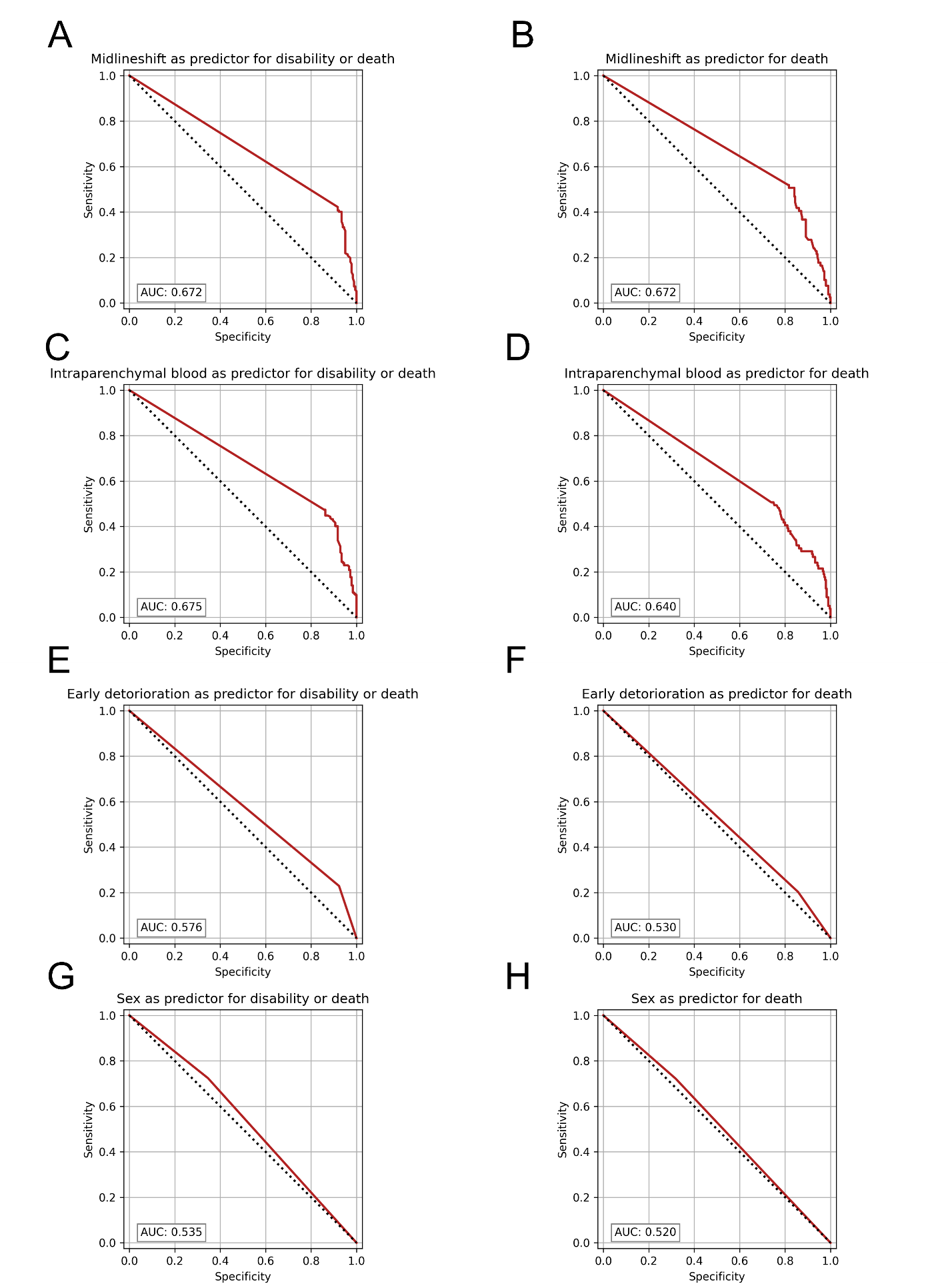
**

**Supplemental Figure 3:**

Predictive value of each EBI-parameter for the outcome (6 months mRS) using receiver operating characteristics (ROC) curves.


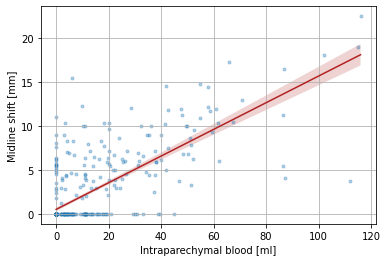


**Supplementary Figure 4:** Collinearity of Midlineshift and intraparenchymal blood volume

| Variables | p-value | Odds ratio | AIC |
| --- | --- | --- | --- |
| WFNS | **< 10^-23^** | 2 | 661.9 |
| Early Ischemia | **< 10^-23^** | 13.5 | 664,3 |
| GCS | **< 10^-22^** | 0.8 | 669.6 |
| Mydriasis | **< 10^-15^** | 7.6 | 693.3 |
| Midlineshift | **< 10^-11^** | 1.2 | 728.6 |
| Age | **< 10^-11^** | 1.1 | 732.0 |
| Intraparenchymal blood | **< 10^-8^** | 1 | 741.2 |
| Fisher grade | **< 10^-4^** | 2.4 | 763.6 |
| Cardiopulmonary resuscitation | **< 10^-4^** | 15.5 | 761.2 |
| mFisher | **< 0.001** | 1.5 | 768.6 |
| Early deterioration | **< 0.001** | 2.4 | 772.9 |
| Sex | 0.17 | 0.7 | 782.5 |
| Aneurysm location | 1.00 | 0 | 781.3 |
| Therapy | 1.00 | 1 | 715.5 |

**Supplementary Table 1:** Univariate Analysis of EBI-Phase parameters

AIC Akaike information criterion, Significant Results are bold.
